# Testosterone Influence on Gene Expression in Lacrimal Glands of Mouse Models of Sjögren Syndrome

**DOI:** 10.1167/iovs.19-26815

**Published:** 2019-05

**Authors:** Mathias Kaurstad Morthen, Sara Tellefsen, Stephen M. Richards, Scott M. Lieberman, Raheleh Rahimi Darabad, Wendy R. Kam, David A. Sullivan

**Affiliations:** 1 Schepens Eye Research Institute of Massachusetts Eye and Ear, Boston, Massachusetts, United States; 2 Department of Medical Biochemistry, Oslo University Hospital/Faculty of Medicine, University of Oslo, Oslo, Norway; 3 Department of Genetics & Evolution, School of Biological Sciences, The University of Adelaide, Adelaide, Australia; 4 Stead Family Department of Pediatrics, Carver College of Medicine, University of Iowa, Iowa City, Iowa, United States; 5 Department of Clinical Anesthesia, Indiana University School of Medicine, Indianapolis, Indiana, United States; 6 Department of Ophthalmology, Harvard Medical School, Boston, Massachusetts, United States

**Keywords:** testosterone, Sjögren syndrome, lacrimal gland, gene expression, MRL/lpr-lpr/lpr mice, nonobese diabetic mice

## Abstract

**Purpose:**

Sjögren syndrome is an autoimmune disorder that occurs almost exclusively in women and is associated with extensive inflammation in lacrimal tissue, an immune-mediated destruction and/or dysfunction of glandular epithelial cells, and a significant decrease in aqueous tear secretion. We discovered that androgens suppress the inflammation in, and enhance the function of, lacrimal glands in female mouse models (e.g., MRL/MpJ-Tnfrsf6^lpr^ [MRL/lpr]) of Sjögren syndrome. In contrast, others have reported that androgens induce an anomalous immunopathology in lacrimal glands of nonobese diabetic/LtJ (NOD) mice. We tested our hypothesis that these hormone actions reflect unique, strain- and tissue-specific effects, which involve significant changes in the expression of immune-related glandular genes.

**Methods:**

Lacrimal glands were obtained from age-matched, adult, female MRL/lpr and NOD mice after treatment with vehicle or testosterone for up to 3 weeks. Tissues were processed for analysis of differentially expressed mRNAs using CodeLink Bioarrays and Affymetrix GeneChips. Data were analyzed with bioinformatics and statistical software.

**Results:**

Testosterone significantly influenced the expression of numerous immune-related genes, ontologies, and Kyoto Encyclopedia of Genes and Genomes (KEGG) pathways in lacrimal glands of MRL/lpr and NOD mice. The nature of this hormone-induced immune response was dependent upon the autoimmune strain, and was not duplicated within lacrimal tissues of nonautoimmune BALB/c mice. The majority of immune-response genes regulated by testosterone were of the inflammatory type.

**Conclusions:**

Our findings support our hypothesis and indicate a major role for the lacrimal gland microenvironment in mediating androgen effects on immune gene expression.

One leading cause of aqueous-deficient dry eye disease (ADDE) in humans is Sjögren syndrome (SS).^1^ This autoimmune disease occurs almost exclusively in women and is associated with an extensive inflammation in the lacrimal gland, immune-mediated destruction and/or dysfunction of glandular epithelial cells, and significant decrease in aqueous tear output.^1^ This sexual dichotomy in SS prevalence has been linked to the more potent immune capability of women,^2^–^4^ as well as to the differential action of sex steroids on the immune system.^5^ Androgens often provide a protective influence and suppress various immunopathologies in SS and other autoimmune diseases. In contrast, estrogens have been implicated in the pathogenesis and/or progression of numerous autoimmune disorders, including SS.^2^,^5^–^7^

Of particular interest, androgen deficiency appears to have an important role in the development of lacrimal gland inflammation and ADDE in SS. Correction of this hormonal deficit, in turn, may have a therapeutic benefit. To explain, androgens are very potent regulators of the lacrimal gland and their action seems to account for many of the sex-related differences that exist in the anatomy, biochemistry, physiology, immunology, and molecular biology of this tissue.^5^ However, androgen levels in women with SS are significantly decreased.^8^–^10^ We hypothesized that this reduction predisposes to lacrimal gland dysfunction, attenuated tear secretion, and ADDE. In support of our hypothesis, we discovered that testosterone administration to female mouse models of SS (e.g., MRL/MpJ-Tnfrsf6^lpr^ [MRL/lpr] and NZB/NZW F1) suppresses inflammation in, and increases the functional activity of, lacrimal tissue.^7^,^11^–^15^ Similarly, topical and/or systemic androgen treatment appears to completely resolve lacrimal gland inflammation in dry eye dogs,^16^,^17^ and to alleviate dry eye signs and symptoms and promote tear flow in SS patients.^5^

The mechanism(s) involved in this androgen-induced suppression of lacrimal gland autoimmune disease in SS remains to be clarified. Our evidence indicates that this hormone action is a unique, tissue-specific effect, which is initiated through androgen binding to specific receptors in lacrimal gland epithelial cells.^7^ In addition, we hypothesize that this androgen interaction then elicits the altered expression and/or activity of immune-related genes in lacrimal tissue, leading to a decrease in immunopathologic lesions and an improvement in glandular function.

To begin to test this hypothesis, we examined the nature and magnitude of testosterone's influence on immune-related gene expression in the autoimmune lacrimal tissues of female MRL/lpr mice after onset of disease. We chose the MRL/lpr strain because, like in humans, the extent of lacrimal and salivary gland inflammation in MRL/lpr mice is far greater in females compared to males,^18^ and is dramatically reduced in response to androgen treatment.^7^,^11^–^14^

For comparative purposes, we also analyzed and compared the androgen impact on immune gene expression in lacrimal glands of female nonobese diabetic/LtJ (NOD) mice after onset of disease. These mice, which are an established model for type-1 insulin-dependent diabetes mellitus,^19^ have been used as a model for Sjögren syndrome^20^–^22^ and, like in humans, have far greater inflammation in the salivary glands of females compared to males.^18^ However, unlike humans, the lacrimal glands of male NOD mice have significantly higher inflammation than those of females.^18^,^23^–^25^ Indeed, orchiectomy of NOD mice attenuates, whereas androgen treatment of castrated NOD males induces, lymphocyte accumulation in their lacrimal glands.^23^ This anomalous hormone effect is mediated through the lacrimal microenvironment^24^ and contrasts with the androgen-induced decrease in inflammation in salivary and pancreatic tissues in these mice.^26^,^27^ Given this background, we hypothesized that androgen exposure will significantly increase the expression and/or activity of immune-related genes in the lacrimal glands of female NOD mice. We also hypothesized that these opposing actions of androgens in female MRL/lpr and NOD lacrimal tissues involve regulation of similar immune-related genes, ontologies, and pathways.

## Materials and Methods

### Animals and Tissue Collections

Adult female MRL/lpr and NOD mice were purchased from the Jackson Laboratories (Bar Harbor, ME, USA). Animals were maintained in constant temperature rooms with fixed light/dark intervals 12 hours in duration. Pellets containing vehicle (cholesterol, methylcellulose, lactose) or testosterone (T; 10 mg) were implanted subcutaneously in MRL/lpr (17.1–18.1 weeks old) and NOD (21 weeks old) mice. The pellets were obtained from Innovative Research of America (Sarasota, FL, USA) and were designed for constant release of placebo (P) or physiologic amounts of androgen (for a male^11^–^14^) for a 3-week period. After 20 to 21 days of treatment, mice (*n* = 7–18 mice/condition) were killed by CO_2_ inhalation and exorbital lacrimal glands were removed for molecular biological procedures. Lacrimal tissue samples were prepared by combining glands from two to six mice/strain/group. Three different sample preparations were made for each treatment (i.e., 4–12 lacrimal glands/sample/treatment/strain) and then processed for analysis of gene expression.

All mouse studies were approved by the institutional animal care and use committee of the Schepens Eye Research Institute and adhered to the Association for Research in Vision and Ophthalmology Statement for the Use of Animals in Ophthalmic and Vision Research.

### Molecular Biological Procedures

To determine the effect of T on lacrimal gland gene expression, total RNA was isolated from lacrimal tissues using TRIzol reagent (Invitrogen, Carlsbad, CA, USA) and purified with RNAqueous spin columns (Ambion, Austin, TX, USA). Lacrimal gland RNA samples were treated with RNase-free DNase (Invitrogen), assessed spectrophotometrically at 260 nm to determine concentration, and examined with a RNA 6000 Nano LabChip and an Agilent 2100 Bioanalyzer (Agilent Technologies, Palo Alto, CA, USA) to verify RNA integrity. The RNA samples were kept at −80°C until further processing.

Gene expression was determined via two different procedures. One involved hybridization of lacrimal gland RNA samples to CodeLink (CL) UniSet Mouse 20K I Bioarrays (*n* ∼ 20,000 genes/array; Amersham Biosciences/GE Healthcare, Piscataway, NJ, USA), according to reported methods.^28^ cDNA was generated from RNA (2 μg) with a CL Expression Assay Reagent Kit (Amersham) and purified with a QIAquick purification kit (Qiagen, Valencia, CA, USA). Samples were dried, and cRNA was made with a CL Expression Assay Reagent Kit (Amersham), recovered with an RNeasy kit (Qiagen), and quantified with an ultraviolet spectrophotometer. Fragmented, biotin-labeled cRNA then was incubated and shaken at 300 rpm on a CL Bioarray at 37°C for 18 hours. Following this time interval, the Bioarray was washed, exposed to streptavidin-Alexa 647, and scanned using ScanArray Express software and a ScanArray Express HT scanner (Packard BioScience, Meriden, CT, USA) with the laser set at 635 nm, laser power at 100%, and photomultiplier tube voltage at 60%. Scanned image files were evaluated using CL image and data analysis software (Amersham), which gave raw and normalized hybridization signal intensities for each array spot. The intensities of the approximately 20,000 spots on the Bioarray image were normalized to a median of 1. Standardized data, with signal intensities >0.50, were analyzed with bioinformatic software (Geospiza, Seattle, WA, USA). This comprehensive software also produced gene ontology, Kyoto Encyclopedia of Genes and Genomes (KEGG) pathway, and *z*-score reports. The ontologies included those related to biological processes, molecular functions, and cellular components, and were organized according to the recommended guidelines of the Gene Ontology Consortium (available in the public domain at http://www.geneontology.org/GO.doc.html).^29^

The second method to determine differential gene expression entailed hybridization of each cRNA (20 μg) sample to a GeneChip Mouse Genome 430A 2.0 Array (Affymetrix [Affy], Santa Clara, CA, USA) according to the manufacturer's protocol. Reagents for the fragmentation and hybridization steps originated from a GeneChip HT One-Cycle Target Labeling and Control Kit, and materials for the washing and staining steps were from a GeneChip HWS kit (Affy). Hybridized GeneChips were scanned with an Affy Model 700 Scanner and expression data files were generated from array images using Affy Microarray Suite 4.0 software. GeneChip data were normalized by choosing the default scaling in the Affy GeneChip operating software, which gives a trimmed mean intensity of 500 for each GeneChip microarray. Standardized data with a quality value of 1.0 then were evaluated with Geospiza GeneSifter software.

As we reported recently,^30^ counts of unique mappings of probes to gene identifications in the CL and Affy arrays demonstrated that there were 15,711 and 13,265 unique genes, respectively, in these arrays. Examination of the intersection of these lists showed that there was an overlap of 11,299 genes.

Gene expression data were evaluated without log transformation and statistical analyses were conducted with Student's *t*-test (2-tailed, unpaired) using the GeneSifter software. Our statistical method was not tailored for multiple comparisons. Genes expressed in the same direction in comparative groups were identified using GenBank accession numbers and a Geospiza intersector program. Data used for these CL and Affy arrays are accessible for free download through the National Center for Biotechnology Information's Gene Expression Omnibus (NCBI GEO) via series accession number GSE5877.

We also compared our results to data from our studies examining the influence of sex in adult MRL/lpr and NOD mice (*n* = 15–18/sex/strain),^30^ and 2 weeks of P or T treatment of nonautoimmune, ovariectomized BALB/c mice (*n* = 5–6 mice/condition/experiment),^31^ on lacrimal gland gene expression. The sex- and hormone-related data are available through the NCBI GEO via series accession numbers GSE5876 and GSE3995, respectively.

## Results

### T Influence on Gene Expression in Lacrimal Glands of Female MRL/lpr and NOD Mice

To determine the effect of androgen treatment on gene expression in lacrimal glands of autoimmune mice, tissues were obtained from female MRL/lpr and NOD mice (*n* = 7–18 mice/strain/treatment) following 20 to 21 days of exposure to P or T. Glands were pooled according to treatment and strain (*n* = 4–12 glands/samples/strain/treatment; *n* = 3 samples/treatment group), processed for isolation of total RNA, and analyzed for differentially expressed mRNAs using CL Bioarrays and Affy GeneChips. Microarray data were evaluated with Geospiza bioinformatics software.

Our results with CL and Affy microarrays showed that testosterone treatment has a significant influence on expression of thousands of genes in lacrimal glands of MRL/lpr and NOD mice (Table 1). Androgen exposure increased (↑) the activity of genes, such as cytochrome P450, family 2, subfamily j, polypeptide 13 (Cyt), and decreased (↓) that of pancreatic lipase–related protein 1 (PL) in both strains (Tables 2, 3). These two genes also are regulated in the same manner in lacrimal tissues of nonautoimmune female BALB/c mice (Cyt = 9.9-fold ↑; PL = 81.1-fold ↓; NCBI GEO GSE3995).^31^

**Table 1:** Effect of T on Gene Expression in Lacrimal Glands of Female MRL/lpr and NOD Mice

**Mouse Strain/Array**	**Genes ↑**	**Genes ↓**	**Total Genes**
MRL/lpr			
CL	1890	1708	3598
Affy	1120	1530	2650
NOD			
CL	1474	2275	3749
Affy	1102	1150	2252

Data were evaluated without log transformation. The expression of listed genes was significantly (*P* < 0.05) up (↑)- or down (↓)-regulated by T treatment.

**Table 2:** T Influence on Gene Expression in Lacrimal Glands of Female MRL/lpr Mice

**Accession #**	**Gene**	**Ratio**	***P*** **Value**	**Ontology**
T>P, CL				
NM_145548	Cytochrome P450, family 2, subfamily j, polypeptide 13	528.0	0.0000	Oxidation-reduction process
NM_010643	Kallikrein 1-related peptidase b24	273.1	0.0000	Proteolysis
NM_001042711.2	Amylase 2, pancreatic	204.6	0.0087	Endohydrolysis
NM_146592	Olfactory receptor 1086	151.5	0.0062	Signal transduction
NM_020277	Transient receptor potential cation channel, subfamily M, member 5	142.2	0.0087	Transport
NM_146805	Olfactory receptor 907	121.9	0.0074	Signal transduction
BB149074	Oxysterol binding protein-like 3	111.5	0.0004	Transport
NM_016672	Dopa decarboxylase	109.6	0.0154	Cellular amino acid metabolic process
T>P, Affy				
NM_020268	Kallikrein 1-related peptidase b27	2894.0	0.0008	Proteolysis
NM_019515	Neuromedin U	656.7	0.0006	Energy homeostasis
NM_017390	Seminal vesicle secretory protein 2	328.6	0.0190	Fertilization
BC016446	Cytochrome P450, family 2, subfamily j, polypeptide 13	310.0	0.0018	Oxidation-reduction process
AK020349	Seminal vesicle secretory protein IV	284.2	0.0002	Fertilization
NM_010643	Kallikrein 1-related peptidase b24	268.0	0.0000	Proteolysis
M16360	Major urinary protein V	222.1	0.0011	Transport
AY079153	Melanocortin 2 receptor accessory protein	150.2	0.0001	Positive regulation of camp biosynthetic process
P>T, CL				
NM_018874	Pancreatic lipase related protein 1	21.0	0.0049	Lipid metabolic process
NM_024427	Tropomyosin 1, α	18.8	0.0017	In utero embryonic development
NM_011105	Polycystin and REJ	15.8	0.0003	Transport
NM_009714	Asialoglycoprotein receptor 1	11.4	0.0024	Endocytosis
AK002477	Plasma membrane proteolipid	10.6	0.0009	Transport
NM_026123	Unc-50 homolog	9.5	0.0002	Protein transport
BC018468	Endoplasmic reticulum lectin 1	9.0	0.0053	ER-associated protein catabolic process
AW492955	doublecortin domain containing 2a	8.0	0.0015	Neuron migration
P>T, Affy				
NM_018874	Pancreatic lipase related protein 1	30.1	0.0008	Lipid metabolic process
M30697	ATP-binding cassette, sub-family B, member 1A	28.2	0.0005	ATP catabolic process
BC005555	Prolactin receptor	22.1	0.0009	Activation of transmembrane receptor protein tyrosine kinase activity
NM_008109	Growth differentiation factor 5	15.6	0.0004	Cell-cell signaling
U09362	Asialoglycoprotein receptor 1	13.0	0.0012	Endocytosis
NM_013669	Synaptosomal-associated protein 91	12.9	0.0036	Establishment or maintenance of cell polarity
AF147785	Lost on transformation protein 1	11.6	0.0043	Regulation of gene expression
BC024580	Carboxymethylenebutenolidase-like	7.5	0.0078	Hydrolase activity

Accession numbers are the sequence identities of gene fragments expressed on the CL and Affy microarrays. These sequences appear in the nucleotide database of the NCBI. Relative ratios were determined by comparing the degree of gene expression in lacrimal glands from P- and T-treated female MRL/lpr mice. Ratios were calculated from nontransformed data.

**Table 3:** T Impact on Gene Expression in Lacrimal Glands of Female NOD Mice

**Accession #**	**Gene**	**Ratio**	***P*** **Value**	**Ontology**
T>P, CL				
NM_010639	Kallikrein 1	216.7	0.0237	Proteolysis
NM_145548	Cytochrome P450, family 2, subfamily j, polypeptide 13	212.2	0.0162	Oxidation-reduction process
NM_010643	Kallikrein 1-related peptidase b24	91.1	0.0221	Proteolysis
NM_010644	Kallikrein 1-related peptidase b26	72.9	0.0237	Proteolysis
BC002033	RAD50 homolog	52.0	0.0050	DNA recombination
NM_008957	Patched homolog 1	48.5	0.0071	Signal transduction
BC012259	Major urinary protein 2	47.9	0.0026	Pheromone binding
AK004371	RAS-like, family 11, member A	42.5	0.0007	GTP catabolic process
T>P, Affy				
BC016446	Cytochrome P450, family 2, subfamily j, polypeptide 13	618.5	0.0125	Oxidation-reduction process
U82380	Submaxillary gland androgen regulated protein 2	361.9	0.0003	Response to toxin
NM_010644	Kallikrein 1-related peptidase b26	247.1	0.0145	Proteolysis
BC026378	Kallikrein 1-related peptidase b1	207.3	0.0004	Proteolysis
NM_133997	Apolipoprotein F	154.3	0.0035	Lipid metabolic process
BC009165	Thyroid hormone responsive SPOT14 homolog	95.6	0.0001	Transcription, DNA-dependent
AB065134	Monooxygenase, DBH-like 2	90.4	0.0114	Catecholamine metabolic process
AY079153	Melanocortin 2 receptor accessory protein	84.2	0.0060	Positive regulation of camp biosynthetic process
P>T, CL				
NM_018874	Pancreatic lipase related protein 1	1877.1	0.0475	Lipid metabolic process
NM_011105	Polycystin and REJ	38.3	0.0001	Transport
AK002477	Plasma membrane proteolipid	33.6	0.0005	Transport
NM_011857	Teneurin-3	31.1	0.0002	Signal transduction
NM_010726	Phytanoyl-CoA hydroxylase	24.7	0.0001	Fatty acid α-oxidation
NM_026754	Unique cartilage matrix-associated protein	24.4	0.0197	Negative regulation of osteoblast differentiation
NM_019752	HtrA serine peptidase 2	24.4	0.0009	Proteolysis
NM_146242	Leucine rich repeat containing 10	24.3	0.0001	Transport
P>T, Affy				
NM_018874	Pancreatic lipase related protein 1	1462.8	0.0059	Lipid metabolic process
AF147785	Lost on transformation protein 1	33.2	0.0002	Regulation of gene expression
BC005555	Prolactin receptor	23.4	0.0095	Activation of transmembrane receptor protein tyrosine kinase activity
NM_010726	Phytanoyl-CoA hydroxylase	21.8	0.0096	Fatty acid α-oxidation
AK014353	KH domain containing, RNA binding, signal transduction associated 3	13.5	0.0000	Transcription, DNA-dependent
BC011209	Major facilitator superfamily domain containing 7C	13.5	0.0019	Transport
AY061807	Calmodulin-like 4	13.1	0.0002	Calcium ion binding
NM_134094	Neurocalcin delta	13.0	0.0009	Calcium-mediated signaling

Genes with known ontologies are listed. Relative ratios were calculated by comparing the degree of gene expression in lacrimal glands from P- and T-treated female NOD mice. Ratios were generated from nontransformed data.

Examples of other genes upregulated in lacrimal glands of MRL/lpr mice, such as oxysterol binding protein-like 3, olfactory receptor 1086, and dopa decarboxylase (Table 2), also were very highly upregulated by 39.4-, 36.8-, and 58.6-fold amounts, respectively, in NOD lacrimal tissues. In contrast, the gene expression for cathepsin S, which is significantly elevated in the tears of Sjögren syndrome patients,^32^ was significantly (*P* < 0.05) decreased by testosterone in female MRL/lpr lacrimal glands (CL = 1.53-fold ↓; Affy = 1.88-fold ↓), but increased by androgen treatment in those of female NOD mice (CL = 3.87-fold ↑; Affy = 3.23-fold ↑). A similar pattern was found for *moesin* gene expression, which was reduced by T in female MRL/lpr lacrimal glands (Affy = 3.19-fold ↓), but increased by androgen exposure in lacrimal tissues of female NOD mice (Affy = 3.39-fold ↑). Other genes were regulated by T in the lacrimal tissue of only one strain (e.g., NOD, spleen tyrosine kinase [Syk]; CL = 3.1-fold ↑).

As we^30^,^33^–^35^ and other investigators^36^–^39^ have discovered, the vast majority of lacrimal gland genes in MRL/lpr and NOD female mice, which were identified as differentially expressed by the CL and Affy microarrays, were unique to each platform. Indeed, as demonstrated in Table 4, only 8.5% to 11.1% (T>P), and 7.3% to 16.8% (P>T) of the regulated genes were found by both microarrays. These data showed that there are significant differences in the ability of these platforms to detect differential gene expression.

**Table 4:** Comparative Gene Expression Between CL and Affy Microarrays

	**Genes T>P**	**Genes P>T**	**Total Genes**
MRL/lpr			
CL			
Unique CL genes, not expressed by Affy	1724	1284	3008
Affy			
Unique Affy genes, not expressed by CL	943	1490	2433
CL vs. Affy			
Genes changed in same direction	143	216	359
Genes changed in opposite direction			16
NOD			
CL			
Unique CL genes, not expressed by Affy	1326	2091	3417
Affy			
Unique Affy genes, not expressed by CL	932	973	1905
CL vs. Affy			
Genes changed in same direction	147	152	299
Genes changed in opposite direction			13

Data were evaluated without log transformation. Genes identified as “unique” were significantly (*P* < 0.05) increased on one, but not the other, microarray platform. The phrase “Genes changed in the same (or opposite) direction” means that the results were significant (*P* < 0.05) on both platforms.

This low concordance in gene identification appears to be due to intrinsic variations in multiple aspects of platform design, as well as to the inherent instability of lists of significantly changed genes based upon *P* value cutoffs.^36^–^40^ The result is that CL and Affy microarrays, both of which have documented accuracy and reproducibility, seem to measure different things.^38^ Most gene expression differences revealed by each platform are thought to be biologically correct, and these variations cannot be attributed to technological differences.^37^,^38^

Comparison of gene expression between the lacrimal glands of P-treated MRL/lpr and NOD mice demonstrated that 587 genes were in common (CL). The alternate comparison (i.e., MRL/lpr, T>P; NOD, T>P) revealed 559 genes in common (CL).

### T Effect on Immune-Related Ontologies in Lacrimal Glands of Female MRL/lpr, NOD and BALB/c Mice

T exerted a significant influence on the expression of a large number of immune-related gene ontologies in the lacrimal glands of female MRL/lpr and NOD mice. Many of these hormone responses were identified by CL and Affy platforms (Tables 5, 6).

**Table 5:** T Downregulation of Immune-Related Gene Ontologies in Lacrimal Glands of Female MRL/lpr Mice

**Ontology**	**CL Genes** ↓	**Affy Genes** ↓	**CL** ***z*****-score**	**Affy** ***z*****-score**
Biological process				
Immune system process	166	130	7.27	3.97
Immune response	90	67	5.45	2.65
Defense response	89		4.73	
Regulation of immune system process	79	68	3.9	2.72
Leukocyte activation	65	54	4.58	3.06
Immune system development	61		2.98	
Hemopoietic or lymphoid organ development	58		3.02	
Lymphocyte activation	55	44	4.11	2.4
Positive regulation of immune system process	55	47	3.33	2.12
Response to cytokine stimulus	47	35	6.4	3.76
Innate immune response	46	35	4.75	2.82
Induction of apoptosis	45		2.82	
Regulation of immune response	43		2.31	
Cytokine production	41		3.03	
Regulation of defense response	41		3.6	
Immune effector process	39		2.56	
Inflammatory response	39		2.51	
Leukocyte differentiation	38		3.13	
T cell activation	38		3.46	
Positive regulation of immune response	37		2.82	
Positive regulation of intracellular protein kinase cascade	36		2.84	
Regulation of leukocyte activation	34	34	2.87	3.2
Regulation of cytokine production	33		2.23	
Regulation of lymphocyte activation	32	30	3.13	2.92
Cellular response to cytokine stimulus	31	25	4.45	2.98
Leukocyte proliferation	31	23	4.19	2.22
Activation of immune response	30	24	2.98	2.03
Lymphocyte proliferation	30	22	4.08	2.08
Positive regulation of defense response	29		3.61	
I-κb kinase/NF-κb cascade	25		3.54	
Positive regulation of leukocyte activation	25	24	3.23	2.96
Positive regulation of lymphocyte activation	24	22	3.42	2.77
Regulation of T-cell activation	24	21	2.91	2.44
Cytokine-mediated signaling pathway	23		2.99	
Immune response-activating signal transduction	23	20	2.24	2.03
Immune response-regulating signaling pathway	23		2.13	
Regulation of leukocyte proliferation	23	20	3.44	2.69
Positive regulation of cytokine production	22		3.22	
Regulation of lymphocyte proliferation	22		3.27	
B-cell activation	21		2.16	
Regulation of innate immune response	21		2.24	
T-cell differentiation	20		2.22	
T-cell proliferation	20		3.26	
Activation of innate immune response	18		2.79	
Cytokine biosynthetic process	15		2.35	
Cytokine metabolic process	15		2.35	
Innate immune response-activating signal transduction	17		2.55	
Leukocyte migration	17		2.04	
Myeloid leukocyte activation	14		2.25	
Myeloid leukocyte differentiation	16		2.4	
Positive regulation of I-κb kinase/NF-κb cascade	18		3.38	
Positive regulation of innate immune response	19		2.29	
Positive regulation of leukocyte proliferation	18		3.56	
Positive regulation of lymphocyte proliferation	17		3.37	
Positive regulation of mononuclear cell proliferation	17		3.31	
Positive regulation of T-cell activation	18	18	3.21	3.29
Regulation of I-κb kinase/NF-κb cascade	19		3.18	
Regulation of inflammatory response	17		2.08	
Regulation of innate immune response		19		2.06
Regulation of lymphocyte proliferation		19		2.5
Regulation of T-cell proliferation	15		2.73	
Toll-like receptor signaling pathway	15		2.15	
Molecular function				
Cytokine binding	16		2.22	
Cytokine receptor activity	10		2.35	
Chemokine receptor binding	9	10.00	2.57	3.23
Antigen binding		8.00		5.16
Chemokine activity	7		2.11	
NF-κb binding	5		2	
Cellular components				
MHC protein complex	5		2.38	

Biological process (≥20 genes/ontology), molecular function (≥5 genes/ontology) and cellular component (≥5 genes/ontology) immune ontologies were identified after the analysis of nontransformed CL and Affy data. These immune ontologies were upregulated in lacrimal gland samples from P-treated female mice, and by extension, downregulated in lacrimal gland tissues from androgen-treated mice. A *z*-score is a statistical rating of the relative expression of genes, and shows how greatly they are over- or underrepresented in a specific gene list.^41^ Positive *z*-scores represent a higher number of genes meeting the criterion than is anticipated by chance, and values >2.0 are significant. CL Genes ↓, number of genes downregulated, as calculated with a CL Bioarray; Affy Genes ↓, number of genes downregulated, as determined with Affy GeneChips; *z*-score, specific score for the down-regulated genes in the CL- and Affy-related tissues.

**Table 6:** T Upregulation of Immune-Related Gene Ontologies in Lacrimal Glands of Female NOD Mice

**Ontology**	**CL Genes ↑**	**Affy Genes ↑**	**CL** ***z*****-Score**	**Affy** ***z*****-Score**
Biological process				
Immune system process	192	137	13.29	9.08
Immune response	127	85	13.54	8.8
Defense response	115	80	10.91	7.13
Regulation of immune system process	111	77	11.05	7.28
Leukocyte activation	87	61	10.52	7.15
Positive regulation of immune system process	86	65	10.98	8.35
Regulation of immune response	77	50	10.88	6.86
Lymphocyte activation	72	53	9.2	6.77
Immune effector process	71	46	11.16	7.07
Immune system development	69	50	6.12	4.13
Innate immune response	62	44	10.06	7.31
Positive regulation of immune response	62	44	10.1	7.34
Cytokine production	59	37	8.52	4.88
Regulation of leukocyte activation	54	37	9.16	6.14
T-cell activation	53	40	8.5	6.65
Inflammatory response	51	30	6.57	2.85
Regulation of cytokine production	51	33	7.85	4.74
Leukocyte differentiation	49	35	7.11	4.95
Regulation of defense response	49	33	6.9	4.27
Regulation of lymphocyte activation	48	34	8.63	6.06
Leukocyte mediated immunity	47	28	9.82	5.53
Activation of immune response	44	34	8.01	6.84
Positive regulation of leukocyte activation	43	30	9.99	6.75
Regulation of immune effector process	43	21	9.15	3.79
Lymphocyte mediated immunity	40	26	9.36	6.05
Positive regulation of lymphocyte activation	40	27	9.75	6.21
Chemotaxis	39	29	3.94	2.61
Response to cytokine stimulus	39	30	5.89	4.52
Adaptive immune response	38	28	8.19	6.22
Adaptive immune response based on somatic recombination of immune receptors built from immunoglobulin	38	28	8.36	6.46
Immune response-regulating signaling pathway	38	29	7.65	6.61
Leukocyte proliferation	38	27	7.5	5.21
Lymphocyte differentiation	38	27	6.89	4.73
Immune response-activating signal transduction	37	29	7.51	6.7
Lymphocyte proliferation	37	27	7.41	5.37
Regulation of T-cell activation	35	26	7.42	5.81
Positive regulation of defense response	32	25	5.68	4.8
Regulation of leukocyte proliferation	32	21	7.54	4.68
B-cell activation	31	25	6.32	5.38
Positive regulation of cytokine production	31		7.33	
Regulation of innate immune response	31	21	6.42	4.33
Regulation of lymphocyte proliferation	31	21	7.39	4.82
Positive regulation of immune effector process	30		9.08	
Positive regulation of T-cell activation	30	22	8.82	6.53
Regulation of leukocyte mediated immunity	30		8.99	
T-cell differentiation	30		6.47	
Negative regulation of immune system process	28	18	5.77	3.55
Cellular response to cytokine stimulus	26	23	4.19	4.15
Positive regulation of leukocyte proliferation	26	15	7.74	3.88
Regulation of adaptive immune response	26		7.9	
Regulation of adaptive immune response based on somatic recombination of immune receptors built from	26		7.99	
T-cell proliferation	26	16	6.44	3.65
Myeloid leukocyte activation	25		7.58	
Positive regulation of innate immune response	25	18	5.28	4.02
Positive regulation of lymphocyte proliferation	25	15	7.58	4.04
Regulation of lymphocyte mediated immunity	25		7.93	
Regulation of lymphocyte differentiation	24		7.77	
B-cell mediated immunity	23		7.08	
Immune response-regulating cell surface receptor signaling pathway	23		8.11	
Antigen processing and presentation	22	20	9.6	9.59
I-κb kinase/NF-κb cascade	22	22	3.68	4.77
Immune response-activating cell surface receptor signaling pathway	22		7.86	
Immunoglobulin mediated immune response	22		6.74	
Regulation of B-cell activation	22		7	
Cell activation involved in immune response	21		5.39	
Cytokine biosynthetic process	21		5.59	
Cytokine metabolic process	21		5.59	
Cytokine-mediated signaling pathway	21	16	3.4	2.5
Leukocyte activation involved in immune response	21		5.39	
Positive regulation of adaptive immune response	21		8.66	
Positive regulation of adaptive immune response based on somatic recombination of immune receptors b	21		8.66	
Leukocyte migration	20		4	
Positive regulation of leukocyte mediated immunity	20		8.4	
Positive regulation of lymphocyte mediated immunity	20		8.4	
Regulation of T-cell proliferation	20		5.67	
Activation of innate immune response	19	17	4.1	4.51
Leukocyte mediated cytotoxicity	19		7.39	
Positive regulation of lymphocyte differentiation	19		8.42	
Innate immune response-activating signal transduction	18	17	3.83	4.62
Positive regulation of B-cell activation	18		7.35	
Regulation of T-cell differentiation	18		6.28	
α-β T-cell activation	17		5.05	
Positive regulation of I-κb kinase/NF-κb cascade	17	13	3.94	3.27
Positive regulation of T-cell proliferation	17		6.39	
Regulation of I-κb kinase/NF-κb cascade	17	16	3.41	3.99
Regulation of inflammatory response	17		2.98	
T-cell differentiation in thymus	17		6.18	
B-cell differentiation	16		5.5	
Leukocyte chemotaxis	16		4.47	
Myeloid leukocyte differentiation	16		3.28	
Positive regulation of T-cell differentiation	16		7.45	
Toll-like receptor signaling pathway	16		3.38	
Negative regulation of cytokine production	14		3.93	
Molecular function				
Cytokine activity	29		4.88	
Cytokine receptor binding	24		3.36	
Cytokine binding	20		4.68	
Chemokine receptor binding	11	8	4.46	3.21
Chemokine activity	10	6	4.66	2.59
Antigen binding	9	10	6.9	8.35
Cytokine receptor activity	8		2.06	
Chemokine binding	6		3.33	
Chemokine receptor activity	6		3.64	
C-C chemokine binding	5		3.48	
C-C chemokine receptor activity	5		3.68	
MHC protein binding	5		5.31	
CCR chemokine receptor binding	4		3.6	
Chemoattractant activity	4		2.9	
MHC class I protein binding	4		4.75	
Cellular components				
MHC protein complex	11	11	7.99	8.96
T cell receptor complex	7		8	
MHC class II protein complex	6	6	6.24	6.98
α-β T-cell receptor complex	5		7.32	
CD40 receptor complex	5		5.3	
Immunological synapse	5		4.11	

Biological process (≥20 genes/ontology), molecular function (≥5 genes/ontology) and cellular component (≥5 genes/ontology) immune ontologies were identified after the evaluation of nontransformed CL and Affy data. CL Genes ↑, number of genes up-regulated, as identified with a CL Bioarray; Affy Genes ↑, number of genes up-regulated, as found with Affy GeneChips; z-score, specific score for the up-regulated genes in the CL and Affy related tissues.

As demonstrated in Table 5, androgen administration downregulated the expression of over 60 immune-associated biological process ontologies (≥20 genes/ontology) in lacrimal tissues of female MRL/lpr mice, including those related to immune system processes, lymphocyte activation, cytokine production, and inflammatory response. In contrast, T increased the expression of all of these same immune ontologies, as well as more, in lacrimal glands of female NOD mice (Table 6). These changes were accompanied by down- and upregulation of immune-related molecular function (e.g., chemokine activity) and cellular component (e.g., MHC protein complex) ontologies (≥5 genes/ontology) in lacrimal tissues of MRL/lpr and NOD mice, respectively.

Some genes represented within these immune ontologies were the same (e.g., MRL/lpr ↓ and NOD ↑: chemokine [C-X-C motif] ligand 9 [Cxcl9], IL-1β, and toll-like receptors 1 and 2 [TLR 1 and 2]), but most were not. For example, T decreased the expression of 96 immune-response genes (CL) in lacrimal glands of MRL/lpr mice (Table 7), but the majority of these genes were different than the 133 genes (CL) upregulated in NOD mouse tissues (Table 8). Despite these differences, the androgen-regulated immune-response genes were predominantly inflammatory in nature. Thus, T downregulated the expression of 41 inflammatory genes in MRL/lpr lacrimal tissues and 23 of these were the same as in Table 7. Further, androgen administration increased the expression of 52 inflammatory genes in NOD lacrimal glands and 36 of these were identical to those in Table 8.

**Table 7:** T-Induced Decrease of Gene Expression in the Immune Response Ontology in Lacrimal Glands From Female MRL/lpr Mice

**Gene**	**CL Ratio**	**Affy Ratio**	**CL** ***P*** **Value**	**Affy** ***P*** **Value**
Indoleamine 2,3-dioxygenase 1	4.55		0.0073	
Guanylate binding protein 2*	4.31	2.63	0.0016	0.0004
Linker for activation of T cells family, member 2*	4.09		0.0024	
Ectonucleotide pyrophosphatase/phosphodiesterase		3.61		0.0033
Synaptic cell adhesion molecule 1		3.52		0.0009
Chemokine (C-X-C motif) ligand 11	3.12		0.0185	
Properdin		3.4		0.0242
Chemokine (C-X-C motif) ligand 16	3.01	2.08	0.0003	0.0377
Histocompatibility 2, class II antigen A, β1		2.98		0.0037
Complement component 3	2.94	2.53	0.0015	0.0052
C-type lectin domain family 2, member d	2.9	1.9	0.0007	0.0407
Toll-like receptor 4, mRNA	2.89		0.0094	
Interleukin 1 receptor accessory protein, transcript variant 2	2.81		0.0021	
Protein tyrosine phosphatase, receptor type, C, transcript variant 2*	2.8	2.05	0.0155	0.0159
Dual specificity phosphatase 6		2.78		0.0066
Chemokine (C-C motif) ligand 6	2.77	2.33	0.0010	0.0304
Interleukin enhancer binding factor 2	2.77	2.94	0.0260	0.0320
Fc receptor, IgG, α chain transporter	2.73		0.0086	
Bmi1 polycomb ring finger oncogene	2.72	2.02	0.0253	0.0106
Immunoglobulin heavy chain complex*	2.71	4	0.0248	0.0033
Ectonucleotide pyrophosphatase/phosphodiesterase 2	2.68		0.0037	
Immunoglobulin joining chain	2.65		0.0001	
Presenilin 1	2.61		0.0069	
Complement component 1, s subcomponent, transcript variant 1	2.58		0.0013	
2-5 oligoadenylate synthetase-like 2*	2.56		0.0051	
Fc receptor, IgE, high affinity I, gamma polypeptide		2.49		0.0037
Guanylate-binding protein 10		2.44		0.0019
SAM and SH3 domain containing 3*	2.42		0.0070	
CD79B antigen*	2.39	2.69	0.0154	0.0050
Vav 1 oncogene*	2.39		0.0314	
Interferon inducible GTPase 1	2.38	1.58	0.0093	0.0438
Chemokine (C-C motif) receptor 2	2.36	1.61	0.0024	0.0404
Protein kinase RICK	2.36		0.0008	
Strain SJL/J small inducible cytokine A4	2.32		0.0032	
Interleukin 33	2.31	2.45	0.0006	0.0114
Tumor necrosis factor, α-induced protein 8-like 2	2.31		0.0405	
Lymphocyte cytosolic protein 1		2.28		0.0204
Serine (or cysteine) peptidase inhibitor, clade G, member 1		2.28		0.0009
Yamaguchi sarcoma viral (v-yes-1) oncogene homolog		2.28		0.0165
Chemokine (C-X-C motif) ligand 13	2.3	2.38	0.0020	0.0036
Guanylate binding protein 3*	2.27		0.0155	
C-type lectin domain family 7, member a	2.24	2.87	0.0015	0.0011
Chemokine (C-X-C motif) ligand 9*	2.22	2.07	0.0260	0.0242
Phospholipid scramblase 1	2.21		0.0070	
Glutathione peroxidase 2	2.19		0.0046	
Annexin A3	2.18	1.89	0.0202	0.0100
A-synuclein	2.15		0.0012	
Guanylate binding protein 4	2.15		0.0072	
Killer cell lectin-like receptor family E member 1	2.15		0.0081	
Interferon induced with helicase C domain 1		2.15		0.0198
Transporter 1, ATP-binding cassette, sub-family B4	2.13		0.0244	
Dual specificity phosphatase 6	2.07		0.0183	
Nuclear receptor subfamily 1, group H, member 3	2.07		0.0093	
Vitronectin	2.07		0.0457	
Toll-like receptor 3	2.06		0.0029	
Syntaxin binding protein 2		2.06		0.0229
CD55 antigen	2.04		0.0146	
Toll-like receptor 2	2.02	3.51	0.0104	0.0035
High mobility group box 1		2.02		0.0012
Guanylate binding protein 6	2		0.0040	

Relative ratios were calculated from CL and Affy data by comparing the degree of gene expression in lacrimal glands from P- versus T-treated female MRL/lpr mice. Listed CL genes were increased ≥2-fold.

* Genes were found to be upregulated in lacrimal glands of female NOD mice treated with T (Table 8).

**Table 8:** T-Stimulated Increase of Gene Expression in the Immune Response Ontology in Lacrimal Glands From Female NOD Mice

**Gene**	**CL Ratio**	**Affy Ratio**	**CL** ***P*** **Value**	**Affy** ***P*** **Value**
Chemokine (C-X-C motif) ligand 10	10.71	4.86	0.0056	0.0183
Forkhead box P3	10.7		0.0007	
MHC I=H-2Kd homolog	10.39		0.0001	
Chemokine (C-X-C motif) ligand 9*	10.01	33.35	0.0066	0.0021
Adenosine A2b receptor	9.81	3.87	0.0145	0.0002
Histocompatibility 2, K1, K region	9.71		0.0000	
Interferon regulatory factor 7	9.33	4.15	0.0198	0.0002
Interleukin 21	8.12		0.0031	
Tripartite motif-containing 25	8.01		0.0205	
Chemokine (C-C motif) ligand 5	7.21	15.38	0.0192	0.0103
Histocompatibility 2, blastocyst	7.2		0.0075	
Chemokine (C-C motif) ligand 8	7.06	7.68	0.0017	0.0004
Lymphotoxin B	6.63	8.35	0.0115	0.0082
Guanylate binding protein 2*	6.54	6.6	0.0204	0.0029
CD86 antigen	6.37	4.34	0.0035	0.0013
Interferon regulatory factor 8, mRNA (cDNA clone MGC:6194 IMAGE:3487214)	6.35	5.34	0.0026	0.0068
CD247 antigen	6.19		0.0036	
Guanylate-binding protein 10	6.12		0.0279	
Complement component 4B	6.05	4.46	0.0054	0.0044
Chemokine (C motif) ligand 1	5.94		0.0247	
Transporter 1, ATP-binding cassette, sub-family B	5.84	7.76	0.0009	0.0000
Chemokine (C-C motif) receptor 7	5.75		0.0411	
Immunity-related GTPase family M member 1	5.72	5.63	0.0051	0.0074
Myxovirus (influenza virus) resistance 2)	5.72		0.0042	
Solute carrier family 11, member 1	5.67		0.0119	
Tumor necrosis factor receptor superfamily, member 1b	5.65		0.0007	
Interferon regulatory factor 8	5.44		0.0001	
Immunity-related GTPase family M member 2	5.14	7.03	0.0091	0.0158
Bone morphogenetic protein 6	5.13		0.0447	
Similar to histocompatibility 2, D region locus 1	5.06	3.94	0.0053	0.0238
Cytochrome b-245, α polypeptide	5.03	5.79	0.0211	0.0092
Guanylate binding protein 3*	4.99	4.51	0.0107	0.0010
Chemokine (C-C motif) ligand 7	4.88		0.0158	
Cell division cycle 2 homolog A	4.87		0.0282	
SAM domain and HD domain, 1	4.81	4.94	0.0045	0.0064
B-2 microglobulin mRNA, segment 1	4.69		0.0012	
CD40 antigen (Cd40), transcript variant 5	4.67		0.0064	
Lymphocyte protein tyrosine kinase	4.66	5.74	0.0039	0.0250
Fc receptor, IgE, high affinity I, gamma polypeptide	4.58		0.0034	
Protein tyrosine phosphatase, receptor type, C, transcript variant 2*	4.54		0.0046	
Immunoglobulin heavy chain complex*	4.46	5.27	0.0058	0.0043
SAM and SH3 domain containing 3*	4.45	2.82	0.0123	0.0054
Arrestin, β2	4.44		0.0013	
2-5 oligoadenylate synthetase 1B	4.39		0.0160	
Fc receptor, IgG, high affinity I	4.38	3.21	0.0300	0.0098
Protein tyrosine phosphatase, nonreceptor type 22	4.38		0.0011	
Histocompatibility 2, Q region locus 1	4.35	3.11	0.0145	0.0005
CD79A antigen	4.3		0.0022	
Leukocyte specific transcript 1	4.23		0.0426	
Lymphocyte antigen 86	4.19		0.0386	
2-5 oligoadenylate synthetase-like 2*	4.14	7.25	0.0139	0.0059
Myxovirus resistance 1	4.1		0.0115	
Complement component 1, q subcomponent, C chain	4.04		0.0011	
CD74 antigen	3.98	4.48	0.0119	0.0003
CD79B antigen*	3.95		0.0497	
Toll-like receptor 1	3.95	3.2	0.0138	0.0130
Transforming growth factor, β1	3.9		0.0276	
SH2 domain protein 1A	3.87		0.0210	
Vav 1 oncogene*	3.84		0.0145	
B-2 microglobulin, segment 1	3.79		0.0002	
Interferon-inducible GTPase-like	3.79		0.0006	
Linker for activation of T cells family, member 2*	3.68		0.0308	
Interferon induced with helicase C domain 1	3.59		0.0162	
Complement component 1, q subcomponent, α polypeptide	3.57		0.0062	
Complement component 3a receptor 1	3.57		0.0025	
Interleukin 3	3.55		0.0146	
Histocompatibility 2, class II antigen A, β1	3.54	3.42	0.0106	0.0243
CD3 antigen, epsilon polypeptide	3.52		0.0171	
Interleukin 18 receptor 1	3.52		0.0062	

Relative ratios were determined from CL and Affy data by comparing the degree of gene expression in lacrimal glands from P- versus T-treated female NOD mice. Listed CL genes were increased ≥3.50-fold.

* Genes were found to be down-regulated in lacrimal glands of female MRL/lpr mice treated with T (Table 7).

Not all immune-related responses to T in the lacrimal glands of female MRL/lpr and NOD mice were opposite. As shown in Table 9, the expression of certain inflammatory genes was down- or upregulated in the same way in both strains.

**Table 9:** Inflammatory Gene Responses That Were Similar in Lacrimal Glands From Female MRL/lpr and NOD Mice

**Gene**	**MRL/lpr Ratio**	**NOD Ratio**	**MRL/lpr** ***P*** **Value**	**NOD** ***P*** **Value**
Downregulation				
Indoleamine 2,3-dioxygenase 1	4.55	3.65	0.0073	0.0172
UDP-Gal:βGlcNAc β 1,4-galactosyltransferase, polypeptide 1	4.52	12.98	0.0006	0.0009
Interleukin 33	2.31	1.84	0.0006	0.0352
Tachykinin 1	2.17	2.54	0.0236	0.0010
Purinergic receptor P2X, ligand-gated ion channel	1.63	1.78	0.0325	0.0255
Adiponectin, C1Q and collagen domain containing	1.46	3.58	0.0101	0.0046
Junction adhesion molecule 3	1.4	1.77	0.0068	0.0136
Upregulation				
TLR4 interactor with leucine rich repeats	30.47	7.29	0.0277	0.0011
Adenosine A2b receptor	22.87	9.81	0.0053	0.0145
Paraneoplastic antigen MA1	16.63	2.91	0.0001	0.0181
Carbohydrate sulfotransferase 2	15.49	2.13	0.0012	0.0130
Forkhead box P3	11.51	10.7	0.0036	0.0007
Nucleotide-binding oligomerization domain containing 2	10.82	2.65	0.0134	0.0004
Toll-like receptor 9	3.45	1.67	0.0125	0.0170
Chemokine (C-C motif) receptor 5	2.25	2.05	0.0045	0.0078
V-rel reticuloendotheliosis viral oncogene homolog A	1.66	3.06	0.0091	0.0033
Transforming growth factor, β1	1.47	3.9	0.0043	0.0276
Regenerating islet-derived 3α	1.42	1.43	0.0207	0.0222

Relative ratios were calculated from CL data by comparing the degree to which gene expression was significantly down- or upregulated by T treatment, relative to that of P, in lacrimal glands of female MRL/lpr and NOD mice.

The modulatory effect of T on immune-related gene expression in the autoimmune mouse lacrimal glands did not reflect an androgen action typically found in lacrimal tissues of a nonautoimmune strain. Indeed, the effect of T on gene ontologies in lacrimal glands of female NOD, compared to female BALB/c, mice showed significant differences. For example, 21 of 22 androgen upregulated biological process ontologies (Affy) in NOD mice (*n* = 479 NOD > BALB/c ontologies) with the highest *z*-scores (*z* = 6.85 – 10.59) were all immune-related. In contrast, only two of the 161 biological process gene ontologies expressed to a greater extent in BALB/c versus NOD mice were immune-associated. Instead, the BALB/c biological process ontologies with the highest *z*-scores were translation elongation (*z* = 11.59), translation (*z* = 9.56) and oxidation-reduction (*z* = 6.87). In the same way, some of the top molecular function and cellular component ontologies in T-treated female NOD mice were immune-related antigen binding (*z* = 8.72), chemokine receptor binding (*z* = 4.63), and MHC protein complex (*z* = 6.64), whereas they were structural constituent of ribosome (*z* = 10.94), mitochondrion (*z* = 12.77) and multiple ontologies related to oxidoreductase activities in androgen-treated female BALB/c mice.

### T Impact on Immune-Related KEGG Pathways in Lacrimal Glands of Female MRL/lpr and NOD Mice

T administration led to a significant decrease in the expression of immune-related KEGG pathways in lacrimal glands of female MRL/lpr mice (Table 10). These included such pathways as chemokine signaling, cytokine-cytokine receptor interaction, and leukocyte transendothelial migration (Table 9). In contrast, T induced a significant increase in the expression of these KEGG pathways, as well as many more, in lacrimal tissues of female NOD mice (Table 11).

**Table 10:** Immune KEGG Pathways Downregulated in Lacrimal Glands by T Administration to Female MRL/lpr Mice

**KEGG Pathway**	**CL Genes ↓**	**Affy Genes ↓**	**CL** ***z*****-Score**	**Affy** ***z*****-Score**
Chemokine signaling pathway	28		2.92	
Cytokine-cytokine receptor interaction	36		2.79	
Toll-like receptor signaling pathway	17		2.47	
B cell receptor signaling pathway	14		2.4	
Leukocyte transendothelial migration	17	19	2.05	2.06
Phagosome	22		2.01	

Immune-related KEGG pathways that were decreased in T-, as compared to P-, treated female MRL/lpr mice are listed.

**Table 11:** T Upregulation of Immune KEGG Pathways in Lacrimal Glands of Female NOD Mice

**KEGG Pathway**	**CL Genes ↑**	**Affy Genes ↑**	**CL** ***z*****-Score**	**Affy** ***z*****-Score**
Natural killer cell mediated cytotoxicity	38	17	8.37	2.47
Graft-versus-host disease	18	12	7.96	4.99
Allograft rejection	17	10	7.41	3.94
Antigen processing and presentation	23	16	7.11	4.83
Leishmaniasis	24		7.03	
Autoimmune thyroid disease	17	10	6.4	2.99
Toll-like receptor signaling pathway	26	16	5.71	3.07
Primary immunodeficiency	13	9	5.46	3.87
Phagosome	32	28	4.97	4.85
Cytokine-cytokine receptor interaction	44		4.78	
Chemokine signaling pathway	34	26	4.67	3.52
T-cell receptor signaling pathway	24		4.54	
Systemic lupus erythematosus	17	14	4.25	3.23
B-cell receptor signaling pathway	18	12	4.21	2.77
Jak-STAT signaling pathway	26		3.66	
Intestinal immune network for IgA production	10		3.31	
Fc gamma R-mediated phagocytosis	16	14	2.77	2.85
Complement and coagulation cascades	14		2.76	
Leukocyte transendothelial migration	17	17	2.17	2.8

Immune-related KEGG pathways that were increased in T-, as compared to P-, treated female NOD mice are listed.

### Comparison Between the Influence of Sex and T on Immune-Related Gene Expression in Lacrimal Glands of MRL/lpr and NOD Mice

Lacrimal glands of female MRL/lpr and male NOD mice, compared to their opposite sexes, contain a significantly greater expression of genes, ontologies, and KEGG pathways related to inflammatory responses, antigen processing, and chemokine signaling.^30^ We hypothesized that many of these immune-related genes, ontologies, and pathways are analogous to those T suppresses in female MRL/lpr, and induces in female NOD mouse lacrimal tissues. To test this hypothesis, we compared the sex and T influence on immune-related gene expression in MRL/lpr and NOD mice. We also compared these findings to genes more highly expressed in inflamed (MRL/lpr female and NOD male) versus noninflamed (MRL/lpr male and NOD female) lacrimal tissues.

As shown in Tables 12 to 14, many immune-related biological process ontologies (e.g., inflammatory response), immune response genes (e.g., *complement component 3*) and chemokine KEGG pathway genes (e.g., *chemokine* [*C-X-C motif*] *ligand 9*) that are influenced by sex and T in lacrimal glands of MRL/lpr and NOD mice are identical. Thus, androgen downregulates multiple immune-related genes that are highly expressed in lacrimal tissues of female MRL/lpr mice, and T upregulates the expression of these immune genes, which typically are expressed in NOD males, in female NOD lacrimal tissues. These regulated genes in Tables 12 to 14 are the same as those more highly expressed in inflamed compared to noninflamed lacrimal glands.

**Table 12:** Sex and T Influence on Immune-Related Gene Ontologies in Lacrimal Glands of Autoimmune Mice

Gene Ontology	**lpr**	**lpr**	**lpr**	**lpr**	**NOD**	**NOD**	**NOD**	**NOD**	**lpr +NOD**	**lpr +NOD**
	F ↑	F ↑ *z*	T ↓	T ↓ *z*	M ↑	M ↑ *z*	T ↑	T ↑ *z*	Infl ↑	Infl ↑ *z*
Immune system process	228	6.26	166	7.27	227	14.62	192	13.29	238	18.47
Immune response	133	5.89	90	5.45	152	15.16	127	13.54	161	18.74
Defense response	141	6.12	89	4.73	134	11.7	115	10.91	139	14.42
Regulation of immune system process	119	4.29	79	3.9	128	11.66	111	11.05	130	13.88
Leukocyte activation	100	5.51	65	4.58	99	10.89	87	10.52	112	15.01
Positive regulation of immune system process	85	3.99	55	3.33	102	12.13	86	10.98	103	14.06
Lymphocyte activation	84	4.84	55	4.11	83	9.72	72	9.2	97	14.08
Regulation of immune response	77	4.38	43	2.31	90	11.75	77	10.88	89	13.18
Immune effector process	74	5.38	39	2.56	81	11.65	71	11.16	84	13.89
Innate immune response	56	3.15	46	4.75	69	10.11	62	10.06	74	12.72
Positive regulation of immune response	59	3.72	37	2.82	75	11.48	62	10.1	74	12.76
T-cell activation	55	3.53	38	3.46	60	8.74	53	8.5	67	11.75
Cytokine production	72	5.12	41	3.03	63	7.94	59	8.52	66	9.93
Regulation of leukocyte activation	56	4.11	34	2.87	58	8.71	54	9.16	62	11
Regulation of lymphocyte activation	51	4.12	32	3.13	54	8.81	48	8.63	60	11.65
Inflammatory response	71	4.8	39	2.51	58	6.75	51	6.57	58	7.99
Regulation of cytokine production	62	4.65	33	2.23	54	7.21	51	7.85	58	9.39
Regulation of defense response	60	3.79	41	3.6	55	6.95	49	6.9	56	8.4
Leukocyte proliferation	47	4.96	31	4.19	43	7.72	38	7.5	55	12.35
Lymphocyte proliferation	46	4.95	30	4.08	43	7.93	37	7.41	55	12.62
Activation of immune response	41	2.52	30	2.98	55	9.56	44	8.01	54	10.58
Response to cytokine stimulus	49	3.34	47	6.4	45	6.21	39	5.89	48	8.08
Positive regulation of defense response	39	3.14	29	3.61	37	6.02	32	5.68	37	7.03

The number of genes (i.e., non–*z*-score columns) and *z*-scores (*z*) were obtained by analyzing comparative CL microarray data from lacrimal glands from female (F) versus male (M) and P-versus T-treated MRL/lpr (lpr) and NOD mice. The sex-related data originate from one of our recent publications.^30^ The last two columns on the right show results obtained by comparing gene expression in inflamed (Infl) versus noninflamed lacrimal tissues, as described in the Results section. Ontologies were significantly (*P* < 0.05) up (↑)- or down (↓)-regulated according to the listed sex and hormone treatment.

**Table 13:** Sex and T Effect on the Expression of Immune Response Genes in Lacrimal Glands of Autoimmune Mice

Gene Ontology	**lpr**	**lpr**	**lpr**	**lpr**	**NOD**	**NOD**	**NOD**	**NOD**	**lpr +NOD**	**lpr +NOD**
	F ↑	F ↑ *P*	T ↓	T ↓ *P*	M ↑	M ↑ *P*	T ↑	T ↑ *P*	Infl ↑	Infl ↑ *P*
Chemokine (C-X-C motif) ligand 9	4.21	0.0053	2.22	0.0260	15.74	0.0000	10.01	0.0066	5.56	0.0000
CD79B antigen	3.56	0.0001	2.39	0.0154	11.31	0.0002	3.95	0.0497	5.49	0.0018
SAM and SH3 domain containing 3	3.34	0.0019	2.42	0.0070	6.26	0.0001	4.45	0.0123	4.03	0.0000
Linker for activation of T cells family, member 2	2.48	0.0136	4.09	0.0024	6.04	0.0005	3.68	0.0308	3.93	0.0000
Vav 1 oncogene	2.78	0.0008	2.39	0.0314	5.52	0.0000	3.84	0.0145	3.48	0.0000
Complement component 3	4.38	0.0050	2.94	0.0015	3.14	0.0050	3.15	0.0109	3.44	0.0000
C-type lectin domain family 7, member a	3.11	0.0025	2.24	0.0015	5.45	0.0024	2.99	0.0177	3.29	0.0000
Immunoglobulin heavy chain complex	5.47	0.0014	2.71	0.0248	8.51	0.0028	4.46	0.0058	3.2	0.0000
Complement component 4B	1.52	0.0361	1.98	0.0147	5.38	0.0053	6.05	0.0054	3.12	0.0000
interleukin 4 receptor, α	4.35	0.0039	1.73	0.0241	3.91	0.0025	2.68	0.0279	3.06	0.0015
Chemokine (C-X-C motif) ligand 16	6.95	0.0116	3.01	0.0003	1.75	0.0142	2.86	0.0175	2.93	0.0000
Histocompatibility 2, class II antigen A, α	2.64	0.0142	1.69	0.0065	4.22	0.0017	3.13	0.0064	2.87	0.0000
Toll-like receptor 1	2.34	0.0083	1.45	0.0456	4.28	0.0094	3.95	0.0138	2.79	0.0000
Histocompatibility 2, class II antigen E β	2.3	0.0429	1.59	0.0439	4.87	0.0001	3.18	0.0151	2.77	0.0000
Toll-like receptor 2	3.31	0.0033	2.02	0.0104	3.04	0.0003	2.65	0.0111	2.71	0.0000
Immunity-related GTPase family M member 2	2.25	0.0229	1.94	0.0132	3.79	0.0005	5.14	0.0091	2.69	0.0000
Chemokine (C-C motif) receptor 2	2.31	0.0063	2.36	0.0024	3.38	0.0028	2.45	0.0050	2.63	0.0000
Phospholipid scramblase 1	3.54	0.0323	2.21	0.0070	2.01	0.0002	2.4	0.0140	2.5	0.0001
Purinergic receptor P2Y, G-protein coupled, 14	2.32	0.0114	1.34	0.0460	3.69	0.0028	3.15	0.0074	2.47	0.0000
Transmembrane protein 173	4.54	0.0033	1.31	0.0113	3.07	0.0007	1.85	0.0383	2.34	0.0000
Complement component 1, s subcomponent	3.55	0.0039	2.58	0.0013	1.73	0.0096	1.4	0.0133	2.18	0.0002
Interleukin 1β	1.64	0.0146	1.57	0.0329	2.72	0.0001	1.69	0.0456	1.89	0.0001

Relative ratios and *P* values were calculated from CL data by comparing the degree of gene expression in lacrimal glands from female versus male, P- versus T-treated, and inflamed versus noninflamed MRL/lpr and NOD mice. The categories, abbreviations, and origin of the sex-related data are described in the legend to Table 11.

**Table 14:** Sex and T Impact on the Expression of Genes in the Chemokine KEGG Pathway in Lacrimal Glands Of Autoimmune Mice

Gene Ontology	**lpr**	**lpr**	**lpr**	**lpr**	**NOD**	**NOD**	**NOD**	**NOD**	**lpr +NOD**	**lpr +NOD**
	F ↑	F ↑ *P*	T ↓	T ↓ *P*	M ↑	M ↑*P*	T ↑	T ↑*P*	Infl ↑	Infl ↑ *P*
Chemokine (C-C motif) receptor 1	3.4	0.0025	2.58	0.0061	6.88	0.0013	6.4	0.0012	5.64	0.0022
Chemokine (C-X-C motif) ligand 9	4.21	0.0053	2.22	0.0260	15.74	0.0000	10.01	0.0066	5.56	0.0000
Chemokine (C-C motif) ligand 19	5.29	0.0047	2.24	0.0225	5.58	0.0001	3.53	0.0365	3.8	0.0000
Vav 1 oncogene	2.78	0.0008	2.39	0.0314	5.52	0.0000	3.84	0.0145	3.48	0.0000
Gardner-Rasheed feline sarcoma viral oncogene homolog	1.49	0.0215	1.57	0.0404	9.32	0.0015	4.63	0.0173	3.24	0.0001
Chemokine (C-X-C motif) ligand 16	6.95	0.0116	3.01	0.0003	1.75	0.0142	2.86	0.0175	2.93	0.0000
Chemokine (C-C motif) receptor 2	2.31	0.0063	2.36	0.0024	3.38	0.0028	2.45	0.0050	2.63	0.0000
Hemopoietic cell kinase	1.55	0.0340	1.51	0.0419	4.74	0.0005	4.74	0.0348	2.56	0.0000
Chemokine (C-X-C motif) receptor 6	2.32	0.0226	2.7	0.0129	3.43	0.0056	1.63	0.0500	2.46	0.0000
Guanine nucleotide binding protein, gamma 10	1.8	0.0022	1.69	0.0063	1.55	0.0279	1.32	0.0479	1.59	0.0001

Relative ratios and *P* values were determined from CL data as explained in the legends to Tables 11 and 12.

## Discussion

Our results showed that T significantly influences the expression of numerous immune-related genes, ontologies, and KEGG pathways in lacrimal glands of MRL/lpr and NOD mice. These genes are associated with processes, such as lymphocyte activation, leukocyte transendothelial migration, antigen binding, chemokine signaling, cytokine production, cytokine-cytokine receptor interaction, MHC protein complex, and the inflammatory response. The nature of this androgen-induced response depends upon the autoimmune strain and is not duplicated within lacrimal tissues of nonautoimmune BALB/c mice. The majority of immune-related genes regulated by T are of the inflammatory type. Our findings indicated the lacrimal gland microenvironment as a key mediator of androgen effects on immune gene expression and the associated immunopathology.

Our study was prompted by our earlier discovery that androgens, but not estrogens, dramatically suppress the inflammation in lacrimal tissues of the female MRL/lpr and NZB/NZW FI mouse models of SS.^11^–^14^ We hypothesized that this androgen effect involves an alteration in the expression and/or activity of immune-related genes, because such genes are critically important in innate and adaptive immune responses.^42^ These genes might also have a major role in promoting the multiple immunosuppressive actions of androgens, including those directly on T cells, monocytes, macrophages, neutrophils, and B cell precursors, and indirectly on peripheral B cells.^43^,^44^ These androgen actions lead to regulation of the maturation, proliferation, migration, and/or function of immune cells; synthesis and secretion of antibodies, cytokines, adhesion molecules, and proto-oncogenes; and expression of autoantigens.^2^,^43^,^44^ A result is that androgens are protective in SS, as well as in other autoimmune diseases, such as systemic lupus erythematosus, multiple sclerosis, and rheumatoid arthritis.^2^,^5^,^6^,^43^

We discovered that testosterone suppresses a wide array of immune-related genes in lacrimal glands of female MRL/lpr mice. The question is whether some of these genes may be intricately involved in helping to mediate testosterone's anti-inflammatory action in this tissue. Possible examples abound. For example, the androgen downregulation of *complement 3*, *Cxcl9*, *moesin*, *IL-1β*, and *TLR2* genes may interfere with the early stages of SS disease development and the triggering of an adaptive immune response in the lacrimal gland.^30^,^45^–^50^ However, if these five genes are important for the androgen-induced downregulation of lacrimal gland inflammation in female MRL/lpr mice, why are these same genes upregulated by androgen treatment in lacrimal tissues of female NOD mice?

Indeed, we found that many of the immune response genes, immune-related biological process ontologies, and chemokine KEGG pathway genes that are influenced by sex and T in lacrimal glands of MRL/lpr and NOD mice are identical. Thus, androgen decreased the expression of multiple immune-related genes in lacrimal tissues of female MRL/lpr mice, and T increased the expression of these immune genes, which are typically expressed in NOD males,^30^ in female NOD lacrimal tissues. We also discovered that many of these regulated genes are the same as those typically highly expressed in inflamed compared to noninflamed lacrimal glands.

Are there specific genes, then, that might be responsible, at least in part, for promoting the anomalous androgen-induced inflammation in NOD lacrimal glands? Possible genes might be those encoding kallikrein 1 and its related peptidases (KLKs) b1, b4, b5, b8, b11, b24, and b26. Testosterone increased the expression of these genes by 8.4- to 216.7-fold amounts in female NOD lacrimal tissues. KLKs constitute a family of serine proteases that are stimulated by androgens in other tissues^51^ and appear to have a significant role in the development and progression of autoimmune diseases.^52^,^53^ KLK protein levels are increased in lacrimal glands in primary SS.^54^,^55^ Further, several KLKs act as autoantigens, and may serve to elicit an autoimmune T-cell response against lacrimal tissue and to cause a decrease in aqueous tear secretion.^54^,^56^–^58^ However, it is unlikely that KLKs are the keys to understanding androgen-immune effects in NOD mice. The reason is that T also increases by 1.7- to 273-fold the gene expression of KLKs b1, b4, b8, b10, b11, b16, b21, b24, b26, and b27 in lacrimal glands of female MRL/lpr mice, and by 38.8-fold the KLK b24 gene activity in female nonautoimmune BALB/c mice.^31^

Another gene that might be responsible for increasing the aberrant androgen-induced inflammation in NOD lacrimal glands is *Syk*. This tyrosine kinase is very much involved in signaling pathways in hematopoietic cells, and also functions within epithelial cells to promote inflammatory responses.^59^,^60^ *Syk* inhibition has been proposed as a potential treatment for SLE and SS.^61^ However, although *Syk* gene expression is increased in the inflamed lacrimal glands of female MRL/lpr mice (NCBI GEO series accession number GSE5876), it is not decreased by androgen treatment in this strain. Consequently, if there is a specific lacrimal gland switch that androgens turn on to induce immunopathology in NOD mice, and turn off to suppress inflammation in MRL/lpr mice, then *Syk* is not that switch.

What, then, is that possible on/off switch? We hypothesized that this switch, which may comprise a single or multiple genes, is triggered by an androgen–androgen receptor interaction within lacrimal gland epithelial cell nuclei. These classical androgen receptors are members of the nuclear receptor superfamily of ligand-inducible transcription factors and mediate the majority of androgen actions throughout the body.^62^,^63^ Following androgen association with its specific receptor, the monomeric, activated androgen-receptor complex binds to androgen response elements in the regulatory region of target genes and, in combination with coactivators and enhancers, regulates gene transcription, and ultimately protein synthesis and tissue function.^62^–^67^

We have shown that androgen receptors are located almost exclusively within acinar and ductal epithelial cell nuclei in lacrimal glands of MRL/lpr mice, and are absent within the extensive lymphocytic populations in these autoimmune tissues.^68^ Moreover, we have found that androgens upregulate the expression of androgen receptor protein in MLR/lpr lacrimal gland epithelia, and this autoregulation is particularly intense in ductal epithelial cells.^68^ Indeed, the highest level of androgen receptor protein in ductal nuclei^68^ is elicited by those androgens that possess the greatest anti-inflammatory activity in MRL/lpr lacrimal tissue.^14^ Given the role of the periductal area in promoting inflammation within the lacrimal gland,^69^ it may be that an androgen-controlled on/off switch exists in ductal epithelial cells. Epithelial cells, in turn, are thought to be the primary cells involved in the initiation and perpetuation of glandular autoimmune reactivity in Sjögren syndrome.^70^,^71^

Consistent with a regulatory role for ductal epithelial cells is the finding that infiltration of lacrimal glands in *AIRE*-deficient NOD mice appears to localize to ductal tissue.^72^ AIRE is a transcription factor and autoimmune regulator that enforces self-tolerance; humans expressing a defective form of this gene develop multiorgan autoimmune disease.^73^ Interestingly, correction of ductal epithelial function also has been shown to correct acinar epithelial function.^74^ This domino effect suggests that ductal cells have an essential role in the pathogenesis of lacrimal gland dysfunction and ultimately aqueous tear film deficiency

Why then is there an aberrant androgen immune response in lacrimal glands of NOD mice? Could this response be related to a genetic alteration in the androgen receptor, or to changes in the hypothalamic-pituitary-adrenal (HPA) axis, or to the diabetes that is characteristic of this strain? Defects in sex steroid receptors have been linked to the onset, progression, and severity, as well as the sex-related prevalence, of a number of autoimmune disorders, including lupus, rheumatoid arthritis, and diabetes.^75^ These defects often are due to gene polymorphisms or alternative splicing and may lead to marked changes in the affinity or specificity of ligand binding, nuclear translocation, receptor dimerization, DNA association, and transcriptional activation.^75^ However, we found that the coding region of androgen receptors in lacrimal glands of NOD and MRL/lpr mice is not defective, but rather normal.^75^ As concerns the HPA axis, we previously discovered that hypophysectomy or anterior pituitary ablation significantly interferes with androgen action on the lacrimal gland.^76^ This lacrimal gland impairment appears to be tissue-specific.^77^ However, although the pituitary has blunted responses in humans with SS,^78^ NOD mice have a hyperactive HPA^79^ and this would not inhibit androgen effects on lacrimal tissue. With regard to diabetes, insulin deficiency is known to attenuate the lacrimal gland response to androgen,^80^ but there is no evidence that this condition would promote a completely opposite immune response to androgens as found in NOD compared to MRL/lpr mice.

As one additional consideration, it has been proposed that a defect in male-specific, lacrimal gland-protective T regulatory cells is the cause of the lacrimal gland inflammation in NOD mice, and is driven by a T regulatory cell-extrinsic factor.^81^ However, given that we were able to induce a striking increase in inflammatory gene expression in lacrimal tissue of NOD female mice, it would seem that androgen action has the key role in this T-cell effector/regulator imbalance.

The androgen-induced up- and downregulation of inflammatory gene expression in NOD and MRL/lpr mice, respectively, appears to be mediated through the lacrimal gland environment. Consistent with this hypothesis are the results of adoptive transfer experiments in NOD mice with severe combined immune deficiency (SCID). These animals lack functional T and B cells and do not suffer autoimmune disease. Transfer of splenocytes or cervical lymph node cells from a female NOD mouse to a male NOD.SCID causes massive inflammatory lesions in the lacrimal gland, whereas transfer of male NOD splenocytes or cervical lymph node cells to a female NOD.SCID does not elicit such lacrimal tissue infiltration.^24^,^81^ Further, the lacrimal gland inflammatory response can be reduced by castration of a male NOD mouse,^23^ and induced by androgen treatment of a female NOD mouse (this study).

It is possible that intracrine steroidogenic enzymes convert androgens in the NOD lacrimal gland into metabolites that act through different mechanisms than testosterone, such as may occur in the brain.^82^ Such byproducts could have aberrant forms, given that unusual androgen metabolites are the key serum biomarkers for dry eye disease.^83^ Alternatively, it is possible that epithelial cells in NOD lacrimal tissue, like human prostate epithelial cells, demonstrate significant plasticity in response to androgens.^84^ Nevertheless, the identity of the microenvironmental switch(es) that translate androgen action into an up- or downregulation of immune-related gene expression in the lacrimal gland remains to be discovered.
